# How Internet of Things responds to the COVID-19 pandemic

**DOI:** 10.7717/peerj-cs.776

**Published:** 2021-11-10

**Authors:** Taher A. Ghaleb, Rasha A. Bin-Thalab, Ghadir AbdulhakimAbdo Abdullah Alselwi

**Affiliations:** 1 School of Electrical Engineering and Computer Science, University of Ottawa, Ottawa, Ontario, Canada; 2 Department of Computer Engineering, Hadhramout University, Hadhramout, Yemen; 3 Faculty of Computer and Information Sciences, Sakarya University, Sakarya, Turkey

**Keywords:** Internet of things, COVID-19, Pandemic, Empirical study

## Abstract

The cornovirus disease (COVID-19) pandemic has had a severe impact on our daily lives. As a result, there has been an increasing demand for technological solutions to overcome such challenges. The Internet of Things (IoT) has recently emerged to improve many aspects of human’s day-to-day activities and routines. IoT makes it easier to follow the safety guidelines and precautions provided by the World Health Organization (WHO). Prior reports have shown that the world nowadays may need more IoT facilities than ever before. However, little is known about the reaction of the IoT community towards defeating the COVID-19 pandemic, technologies being used, solutions being provided, and how our societies perceive the IoT means available to them. In this paper, we conduct an empirical study to investigate the IoT response to the COVID-19 pandemic. In particular, we study the characteristics of the IoT solutions hosted on a large online IoT community (*i.e.*, Hackster.io) throughout the year of 2020. The study: (a) explores the proportion, types, and nations of IoT solutions/engineers that contributed to defeating COVID-19, (b) characterizes the complexity of COVID-19 IoT solutions, and (c) identifies how IoT solutions are perceived by the surrounding community. Our results indicate that IoT engineers have been actively working towards providing solutions to help their societies, especially in the most affected nations. Our findings (i) provide insights into the aspects IoT practitioners need to pay more attention to when developing IoT solutions for COVID-19 and to (ii) outlines the common IoT solutions and technologies available to humans to deal with the current challenges.

## Introduction

In *December* 2019, the first coronavirus-infected case was recorded in Wuhan, Hubei Province, China. Then, the coronavirus has started to spread all over the world, contributing to a global crisis (World Health Organization, 2020). COVID-19 was confirmed by the World Health Organization (WHO) as a pandemic on *March* 11th, 2020 (World Health Organization, 2020) after many countries have been affected. The widespread of COVID-19 has resulted in immense human-related problems in terms of the environment, health (Chaudhury et al., 2017), economy, society, and education (Dey, Roy & Das, 2016). Most countries have followed strict procedures to restrain disease transmission by monitoring symptoms, treating patients, quarantining suspicious individuals through tracing, limiting the crowd, and isolation fully or partly. The severe impact of the COVID-19 pandemic has led to freezing most aspects of our daily lives. As a result, there has been an increasing demand for technological solutions to overcome such challenges. In response, researchers from various fields, including science and engineering, have reacted to meet community demands by utilizing technology to provide medical services, instructions, tools for disinfection, and track information and updates about COVID-19.

The Internet of Things (IoT) is one of the technologies that recently emerged to simplify our day-to-day activities and routines. IoT makes it easier to follow the safety guidelines and precautions provided by WHO. Besides health services (Javaid et al., 2020), IoT supports a scalable network that has the capacity to support massive volumes of sensor-collected data to be used by various applications to tackle COVID-19. The world nowadays may need more IoT facilities than ever before. Prior research has shown that IoT applications can play a significant role in the battle against the COVID-19 pandemic (Singh et al., 2020).

However, little is known about how the IoT community is reacting towards defeating the COVID-19 pandemic, the technologies being used, the solutions being provided, and how our society perceives the COVID-19 related IoT projects available to them. In this paper, we conduct an empirical study to investigate the IoT response to the COVID-19 pandemic. We study the characteristics of the currently developed IoT solutions in online IoT communities throughout the year of 2020. In particular, we collect data about COVID-19 IoT projects from Hackster.io (https://www.hackster.io), a large and active IoT development community. We perform analyses on the differences between the COVID-19 and non-COVID-19 IoT solutions in terms of activity, complexity, and popularity.

**Research questions.** This paper conducts an empirical study of the IoT projects for COVID-19  by addressing the following research questions:

***RQ_1_: How active is the IoT community in responding to the COVID-19 pandemic?*** The COVID-19 pandemic is evolving, spreading over the world, and has tremendously affected the lives of our society. Little is known, however, on whether the technology has responded to the pandemic. Our investigation of 946 COVID-19 IoT projects reveals that IoT engineers have been actively working towards providing solutions to help their society. Our results motivate practitioners of other technological fields to act upon the spread of the coronavirus.
***RQ_2_: What aspects of COVID-19 do IoT projects address?*** Little is known about the types of solutions IoT engineers to fight the COVID-19 pandemic. Two of the co-authors performed independent manual analyses of a statistically significant random sample of 274 COVID-19 IoT projects in our dataset to discover what aspects IoT solutions address. We identified four key categories of IoT projects that address COVID-19, namely *Protection*, *Diagnosis*, *Tracking*, and *lockdown-related* projects. The majority of COVID-19 IoT projects in our sample focus on protecting and providing a safe environment to the society. Our findings increase the awareness of society about the IoT solutions available to help them during such a challenging time.
***RQ_3_: How complex are COVID-19 IoT projects?*** Despite the technological advances, responding to the COVID-19 pandemic may require sophisticated infrastructure to effectively mitigate the spread of the coronavirus. We studied the complexity of COVID-19 IoT projects by analyzing the degree of complexity of COVID-19 IoT projects and compare them with unrelated COVID-19 projects. We observed that developing COVID-19 IoT projects may require little expertise but can be costly and time-consuming to reproduce. Our results motivate IoT practitioners to join the battle against COVID-19, since it might just require a little determination.
***RQ_4_: Is the IoT response to the COVID-19 pandemic nation-dependent?*** The impact of the COVID-19 pandemic has been different from one nation to another. Hence, we investigate whether the impact of the pandemic on a certain country yields an analogous impact on the IoT technology. We performed a nation-wise analysis of COVID-19 IoT projects. We observed that the leading countries in developing IoT solutions for COVID-19 are India, the USA, the UK, and China with 143, 69, 26, and 17 projects, respectively. Moreover, we observed that there is a significant (positive) correlation between the number of COVID-19 IoT projects and the number of reported COVID-19 infected cases of the countries in our dataset. This finding suggests that the more a country is affected by the pandemic, the more productive IoT engineers become to develop healthcare solutions.
***RQ_5_: How popular are COVID-19 IoT projects?*** Despite being actively published, it is unclear whether COVID-19 IoT projects receive satisfactory attention by community members. To study the popularity of COVID-19 IoT projects, we analyzed the average number of views and likes of the projects in comparison with the other IoT projects published in the same period. We observed that COVID-19 IoT projects have 2*x* more views and 3*x* more likes than any other IoT projects. Our results indicate that the developed COVID-19 IoT project are meeting the community expectations of being useful to overcome the pandemic.
***RQ_6_: What technologies do COVID-19 IoT projects use in common?*** IoT adopts various software and hardware technologies and platforms. We explored the most prominent technologies used in COVID-19 IoT projects by analyzing the channels and tags associated with each project in our dataset. We observed that the most commonly used hardware technologies/platforms by COVID-19 IoT projects are not far from those of non-COVID-19 IoT projects. Our results encourage the IoT industry to mass-produce their products and export them to more affected nations to broaden the adoption of IoT solution in healthcare systems.

**Paper organization.** The rest of this paper is organized as follows. ‘Background’ gives background on IoT, Hackster.io, and pandemic. ‘Materials & Methods’ describes the methods and materials of our study. ‘Results’ discusses the findings of our empirical study. ‘Discussion’ discusses the implications of our findings. ‘Related work’ presents the literature related to our study. ‘Threats to validity’ lists the threats to the validity of our findings. Finally, ‘Conclusion’ concludes the paper and gives recommendations for future work.

## Background

This section presents background about IoT, Hackster.io, and pandemic-related concepts.

### Internet of Things (IoT)

The Internet of Things (IoT) is a network of physical objects involving smart sensors to identify and interface with their environment and to collect and share knowledge to make life easier. IoT is an application of Artificial Intelligence (AI). The term ‘*Things*’ may refer to any object that has a key to turn on and off. IoT allows devices and machines to communicate with each other *via* the Internet without human intervention. Things can analyze and exchange data to make decisions based on the way they are programmed to. The IoT architecture constitutes several pillars to bridge the gap between the real world and the virtual world (Mattern & Floerkemeier, 2010). Such pillars may include communication, addressability, identification, sensing, actuation, embedded systems, localization, and user interfaces.

The IoT technology is not a new terminology. For example, Automated Teller Machines (ATMs), which allow people to withdraw money from their bank accounts, are of the early examples of IoT artifacts. IoT technologies have been online since 1974. However, nowadays, IoT has been massively due to its availability, affordability, and scalability. IoT systems are now widespread, affordable, and readily replaceable. The rising Internet speeds continue to reduce the costs of IoT research and development. IoT systems are scalable, expandable, and affordable. The cost of sensors has dramatically declined, which makes it available in everyone’s hands. According to the International Data Corporation(IDC) (https://www.idc.com/getdoc.jsp?containerId=IDC_P24793), there will be a market size of $1.1 trillion with billions of devices connected throughout the IoT ecosystem by 2023. However, based on Li, Da Xu & Zhao (2015) and Botta et al. (2014), the heterogeneity and immature standardization of IoT systems increase the complexity of developing IoT systems. Hence, it is important for IoT engineers to share their expertise with other IoT practitioners to allow producing more IoT solutions for our societies.

### Online IoT communities

There are various online communities for both(a) beginners to learn about hardware and IoT development and(b) professionals to share their experiences in IoT. These communities allow IoT engineers to communicate with and learn from each other. Examples of these websites are Hackster.io (https://www.hackster.io), I nstructables (https://www.instructables.com), H ackADay (https://hackaday.com), and H ackr.io. (https://Hackr.io). In this paper, we use Hackster.io as the main source for our study.

**Hackster.io**: Hackster.io is one of the largest and most active IoT development communities. As of *June* 2021, Hackster.io has over 1.6 million community members, with over 20*k* of them are professional engineers (*i.e.,* having at least one published project). Hackster.io allows the community users to access the different resources about the IoT community (https://www.hackster.io/channels), including platforms, communities, and topics.

**IoT platforms**: A platform on Hackster.io represents a group of products that share common hardware and software features. A platform can be a company (*e.g.*, AT&T and Panasonic), a major hardware component (*e.g.*, Arduino and Raspberry Pi), an operating system (*e.g.*, Android), or a cloud backend (*e.g.*, Amazon Web Service). A project can belong to more than one platform. For example, the *COVID-19*: Hand Disinfection Machine (https://www.hackster.io/hivwolf/covid-19-hand-disinfection-machine-f04150) project belongs to platforms, namely *Autodesk* and *Seed*, in addition to three topics, namely *COVID-19*, *Robotics*, and *Home Automation*.

**IoT projects**: Hackster.io hosts over 26*k* IoT projects. Each project on Hackster.io maintains a web page that contains the information and resources related to that project, such as the title, description, team, difficulty level, instructions, estimated replication time, views count, respects count, hardware components, hand tools, software applications, schematics, code, related channels and tags.

**IoT community members**: Every user on Hackster.io maintains an own page that shows the personal and professional profile. Each web page contains information about the projects, followers, followings, tools, platforms, awards, and channels of the community users. In addition, users can add short biographies about themselves to show their interests and skills. Hackster.io maintains a history of all the activities (https://www.hackster.io/dixon415/activity) performed by every community user.

### Pandemic

Pandemic is defined as large-scale infectious disease outbreaks (Madhav et al., 2017). Viruses spread in a wide geographical area and can dramatically increase morbidity and mortality rates and cause significant economic, social, and political disruption. The most common way to minimize the spread of a virus is social distancing (Anderson et al., 2020; Rebmann, 2014), which aims for physical isolation of individuals in the community. Nations must take a set of precautions to achieve social distancing including lockdowns and stay and work from home (Anderson et al., 2020; Rebmann, 2014). The response of individuals to precautionary procedures may be different based on the extent of their appreciation of the risks of the epidemic situation and their confidence in health reports (Yong & Lemyre, 2019). Assessing these risks depends on the psychological factor of the individuals and the ability to quantitatively measure risks. Likewise, one’s view of those around them, their friends and relatives, and what is the belief of who is responsible for reducing the risks: the individual or the government.

Prior research on previous epidemics, including Ebola and swine flu, has shown profound and diverse negative psychological and social consequences (Al Najjar et al., 2016). Popular psychological reactions include virus contracts and ailments if they are affected, family separation and isolation, and even feelings of shame. A study performed by Lu et al. (2020) explained the prevalence of fear within a month of the outbreak of new COVID-19 in the most affected regions of China. The results of the study indicated that 7% of the sample participants experienced this feeling and the most symptoms were in women.

## Materials & Methods

This section presents the experimental setup of our empirical study. We explain how we collect and prepare the data for our studied RQs.

### Data collection

Our study is based on data collected from Hackster.io, an online community of IoT engineers and practitioners for learning, research, programming, hardware development, and sharing experience. Hackster.io hosts a large number of IoT projects, ranging from demonstrations to advanced systems. Hackster.io is different from other websites (*e.g.*, Instructables (https://www.instructables.com) and HackADay (https://hackaday.com) in the sense that it is more dedicated to complete projects that are developed to achieve a specific job. The other websites are too broad in the sense that IoT engineers and companies can even publish hardware and software tools or training courses in addition to IoT projects. In addition, unlike other websites, Hackster.io provides additional information for the IoT community to distinguish between good and bad projects, such as showing the estimated time to reproduce a project, views/likes of a project, difficulty level of the project, and the amount of instructions provided by project owners.

We build a crawler that collects data related to all IoT projects that are hosted by Hackster.io. Each project is associated with meta-attributes, such as its *link*, *title*, *description*, *tags*, *connected channel*, *hardware items*, and *story*. Considering that we are interested in studying the role of IoT amid the pandemic, we select the projects published between *January* 1st, 2020 and *December* 31st, 2020. This selection results in 6,493 projects.

### Identification of COVID-19 projects

We differentiate between projects that address the COVID-19 pandemic from the projects published within the defined time frame but are unrelated to the COVID-19. To this end, we define a set of terms that can distinguish COVID-19 projects from other projects. We use various online resources to identify keywords associated with COVID-19 (https://www.tmc.edu/news/2020/05/covid-19-crisis-catalog-a-glossary-of-terms, https://zenodo.org/record/3819464#.YOytbOhKjZR) to develop an initial set of keywords that are likely to be related to COVID-19, such as ‘covid’, ‘coivd19’, ‘pandemic’, ‘corona virus’, ‘lockdown’, ‘frontline’, ‘distance’, and ‘temperature’. We use a stemming approach to allow searching for word variants (Xu & Croft, 1998). We manually analyze the web pages of a sample of the obtained projects. The manual analysis was performed by two of the co-authors independently using a statistically significant random sample of 274 out of 946 projects (a confidence level of 95% and a confidence interval of ±5%). As a result, we exclude keywords that are too general (*i.e.,* exist in projects that are unrelated to COVID-19. For example, the keyword ‘mask’ can be associated with projects on Halloween masks or masking tapes. In addition, through our manual analysis, we are able to identify more COVID-19-specific keywords. We repeat this process several times until we produce a set of keywords that are highly related to COVID-19 projects only. These keywords are: ‘COVID’, ‘COVID19’, ‘COVID-19’, ‘corona’, ‘coronavirus’, ‘pandemic’, ‘epidemic’, ‘sars-cov-2’, ‘social distancing’, ‘medical ventilator’, and ‘lockdown’. We search for such keywords in the title, description, tags, channels, and story of each project published within the defined time frame. As a result, we obtain 946 IoT projects that are related to COVID-19.

## Results

In this section, we discuss the motivation, approach, and findings of each of our research questions.

### RQ_1_ : How active is the IoT community in responding to the COVID-19 pandemic?

**Motivation.** The COVID-19 pandemic is emerging, rapidly spreading, and has tremendously affected the lives of our societies. Governments and health officials have already responded to the threats of the pandemic and proposed strict measures to fight the coronavirus. Yet, little is known about the role of technology in this matter. In this RQ, we aim to uncover the effort invested by IoT engineers to address the ongoing pandemic and act towards providing safe environments to their communities. It is important for the surrounding community to recognize what is being built for them and how to make use of technological advances. We address this RQ by studying (i) the proportion of COVID-19 IoT projects and IoT engineers and (ii) the publication evolution of COVID-19 IoT projects in online communities. The approaches and results of this RQ are as follows.

### RQ_1.1_ : What is the proportion of COVID-19 IoT projects and IoT engineers?

**Approach.** We compute the ratio of COVID-19 IoT projects over the non-COVID-19 IoT projects during the year of 2020 using the identification approach described in Section 3. In particular, we count the number of (COVID-19 and non-COVID-19) IoT projects for each month from *January* to *December* of 2020, all inclusive. In addition, we compare the ratio of COVID-19 IoT projects with the other health/medical projects published in Hackster.io during 2020. To do this, we collect the channels associated with each project. Our manual analysis reveals seven health/medical-related channels and tags, namely *Health*, *Medical devices*, *Healthcare*, *Fitness*, *Medical*, *Wellness*, and *Medicine*. Then, we count the number of projects that belong to any of those channels. Moreover, we collect information about IoT engineers who contributed to developing COVID-19 IoT projects. We analyze whether those IoT engineers have only focused on developing IoT solutions or participated in other non-COVID-19 projects.
**Observation.** ***COVID-19 IoT projects represent* 54% *of the 2020 projects that address health and medical concerns, and* 14.56% *overall.*** We observe that the majority (*i.e.,*  54%) of the projects assigned to health/medical-related channels are attributed to COVID-19. Moreover, we identify 4,202 unique project owners (*i.e.,* IoT engineers) among all IoT projects in our dataset. 1,081 (*i.e.,* 26%) of the IoT engineers contributed to developing COVID-19 projects.

### RQ_1.2_ : What is the evolution trend of developing COVID-19 IoT projects?

**Figure 1:**
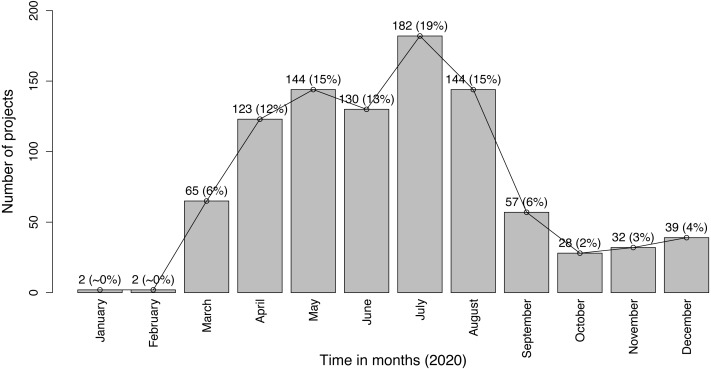
Bar chart of COVID-19 IoT projects from January to December 2020.

**Approach.** To study how the development of COVID-19 IoT projects has been evolving since the coronavirus occurred, we perform a month-by-month analysis of the projects. We collect the date of publication of each COVID-19 IoT project provided by Hackster.io. Then, we compute the ratio of projects for each month (from *January* to *December*, inclusive). We use a bar chart to plot the number and ratio of COVID-19 IoT projects for the twelve months.
**Observation.** ***COVID-19 IoT projects started to rise in March 2020, reaching its maximum (19%) in July 2020.*** Figure 1 shows the number and ratio of COVID-19 IoT projects in 2020. We observe a noticeable increase in the number of projects starting in March, which indicates that IoT engineers started to act towards developing IoT solutions to address COVID-19 when it was characterized as a pandemic by WHO (https://www.who.int/dg/speeches/detail/who-director-general-s-opening-remarks-at-the-media-briefing-on-COVID-19---11-march-2020). In addition, the number of projects continued to increase in *April* and *May*, While the increase was lower in *June* compared to *May*. The number of COVID-19 IoT projects reached its peak in *July*. According to WHO reports, the coronavirus travel and lockdown restrictions began to decline globally (*e.g.*, in Europe (https://www.bbc.com/news/world-europe-52978327) and North America (https://www.cnn.com/world/live-news/coronavirus-pandemic-06-08-20-intl/h_03d1626af89603e81f009995d0814a45) in *June*2020.
  **Conclusion.** Our results indicate that IoT engineers get motivated and become more productive when the society surrounding them becomes more prone to environmental or health issues. This encourages engineers in other technological sectors (*e.g.*, software engineers) to respond to the threats of the virus.

### RQ_2_ : What aspects of COVID-19 do IoT projects address?

**Figure 2:**
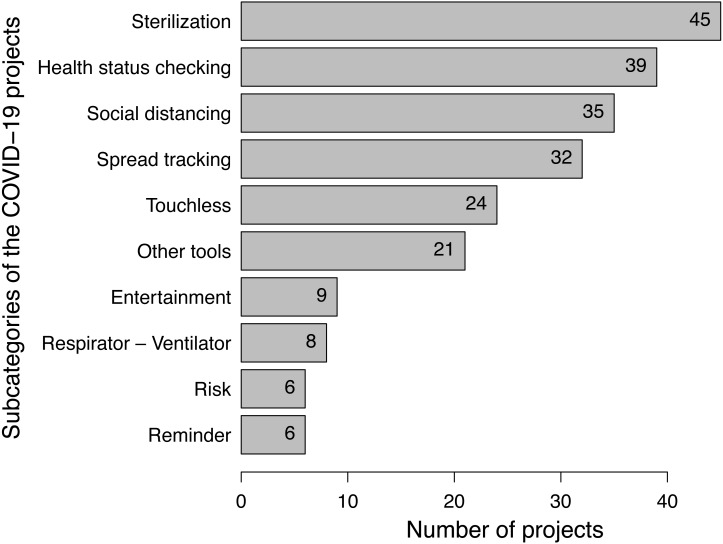
Top 10 sub-categories of C ovid-19 IoT projects.

**Motivation.** RQ1 suggests that IoT engineers have been active to develop IoT solutions to address the COVID-19 pandemic. Still, the type of IoT projects being developed and the COVID-19 aspects they address remains unknown. In this RQ, we study the categories of the COVID-19 IoT projects in our dataset. Findings of this RQ helps to understand whether the developed projects address real problems and are useful to fight the coronavirus.
**Approach.** To categorize COVID-19 IoT projects, we perform a thematic analysis (Fereday & Muir-Cochrane, 2006) to manually identify the aspects (*i.e.,* categories) of COVID-19 issues that IoT projects address. Two of the co-authors (*i.e.,* coders) perform independent manual analyses using a statistically significant random sample of 274 out of 946 projects (a confidence level of 95% and a confidence interval of ±5%) using the formula of Cochran (1977). This means that our categorization of COVID-19 IoT projects is 95% certain with an error margin of plus or minus 5%. We use open coding (Corbin & Strauss, 1990) to produce an initial set of categories of COVID-19 IoT projects. We initially use words that are commonly connected to the coronavirus. (https://www.dictionary.com/e/coronavirus-words, https://www.dictionary.com/e/s/new-words-we-created-because-of-coronavirus). We search for such words in our sampled projects to give us an idea of what each project addresses. Then, each coder manually reviews the title, description, channels, tags, and story of each project to allow assigning appropriate labels. To verify whether the textual description of a project is misleading, we analyze the comments raised by community members to see if someone is raising an issue regarding false information about that project. We were unable to analyze the code of projects, since (a) not all projects have code associated with hardware devices, (b) some projects do not share their code, and (c) the code could be written using different programming languages. Each project is assigned two labels: a main category and a subcategory. After project labeling is finishing, we use Cohen’s *kappa* inter-rater agreement statistic (Cohen, 1960) to measure how reliable is the manually assigned labels by the two authors. We obtain a strong inter-rater agreement (*i.e.,*  *k* = 0.89) with a strong observed agreement of 0.94 between the main categories of projects assigned by the two coders. We also obtain a strong inter-rater agreement (*i.e.,*  *k* = 0.82) with a strong observed agreement of 0.84 between the subcategories of the two coders. Finally, all authors meet to resolve any disagreements in categories. A taxonomy of all categories and subcategories of COVID-19 IoT projects can be found in the supplementary materials.
• **Observation.** ***IoT projects address four aspects of COVID-19, including protection, tracking, diagnosis, and lockdown***. Our manual analysis of COVID-19 IoT projects reveals four main categories of the sampled COVID-19 IoT projects. We observe that some projects may belong to two different categories at the same time. We describe each of the identified categories in the following.
  – **Protection (54%–150 projects):** The main goal of projects of this category is to prevent the spread of coronavirus and protect individuals from getting infected by the coronavirus by establishing different applications to provide precautions as recommended by WHO. We find that *Protection* is the most common category of IoT projects that respond to the COVID-19 pandemic. This result indicates that IoT engineers take the protection of individuals as a key concern, especially that during the year of 2020, there were no effective or proven treatment or vaccines for COVID-19. Protection application include *sterilization*, *vaccination, touchless*, *social distancing*, *mask*, *home automation*, *check and purify air*, *respirator/ventilator*, *frontline worker*, and *reminder for precautions* (*e.g.*, wash your hands or do not touch you face).
  – **Tracking (14%–40 projects):** Tracking is the process of following the course or trail of (someone or something) to locate them or to record their location at various points (https://www.lexico.com/definition/track). This category constitutes 14% of COVID-19 IoT projects. The subcategories of tracking are concerned with tracking the spread of coronavirus, such as recording statistics (internationally or locally), tracing patients (indoors or outdoors), mapping outbreaks, identifying emerging infectious chains, and reporting fake news. Tracking also is used to recognize the faces of people using face recognition techniques (with and without wearing masks or face coverings). Besides, tracking projects may track the number of individuals in a specific area for limiting the crowds.
  – **Diagnosis (14%–40 projects):** The term ‘*diagnosis*’ refers to examining signs or symptoms to identify the nature of an illness or any other problems (https://www.lexico.com/definition/diagnosis). The diagnosis category constitutes IoT projects that aim to identify whether a person is COVID-19 infected by observing signs and symptoms associated with COVID-19 (https://www.cdc.gov/coronavirus/2019-ncov/symptoms-testing/symptoms.html). It is important to diagnose people or patients by checking the status of their health, such as *measuring temperature or pulse oximeter* and *observing other symptoms*. A project may use Machine Learning and AI techniques to enable predicting whether a patient is infected by the coronavirus (https://www.hackster.io/jrsylvester2000/dynamic-face-recognition-based-entry-and-exit-system-03d662).
  – **Lockdown-related (10%–30 projects):** There are projects in our sampled dataset that are not directly related to handling COVID-19 in particular. Instead, projects of this category are either developed as a result of the free time imposed by COVID-19 or to help those people who are in self-isolation or lockdown due to COVID-19. IoT engineers have decided to invest their free time while staying at home to develop IoT solutions that may or may not be directly related to COVID-19. For example, the owner of a *Automated Aeroponic System* https://www.hackster.io/alexch03/automated-aeroponic-system-wifi-remoted-b5eaea is grateful for the lockdown that gave him the chance to develop his first robotic project. We find that projects of this category may address entertainment, work from home, call for help, and indoor training activities.
  – **Overlapping (5%–14 projects):** It is not uncommon to develop projects that address two different aspects of the coronavirus, *i.e.,* overlap between two different categories (*e.g.*, diagnosis and tracking). For example, one project aims to detect temperature and to give a bell alert if there is a high temperature for visitors. https://www.hackster.io/roni-bandini/covid-19-coronavirus-doorbell-114b3f
  * **Subcategories:** Figure 2 shows the top-10 subcategories. We observe similarities with the main categories in which *sterilization* as a subcategory of the protection category reaches 16% (45 projects), followed by the *health status checking* as a subcategory of the diagnosis category at 14% (39 projects), then the *social distancing* from the protection category at 13% (35 projects), and finally the *spread tracking* from the tracking category at 12% (32 projects).
    **Conclusion.** The goal of project categorization is to figure out what areas IoT engineers handled in the battle against COVID-19 and what other areas still need to address. This result shows four main topics are covered: protection, tracking, diagnosis, and lockdown-related projects.

### RQ_3_ : How complex are COVID-19 IoT projects?

**Figure 3:**
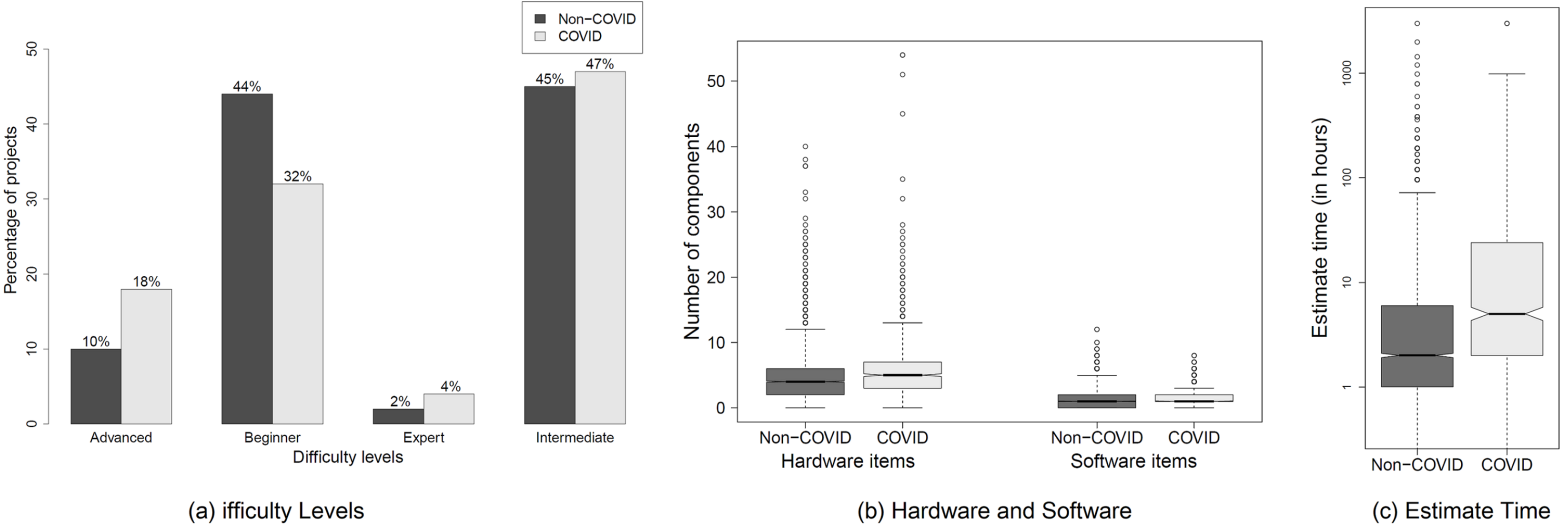
Characteristics of C ovid-19  and non-C ovid-19 IoT projects.

**Motivation.** Despite the technological advances, responding to the COVID-19 pandemic may require sophisticated infrastructure to effectively mitigate the spread of coronavirus. In this RQ, we study the complexity of COVID-19 IoT projects by analyzing the resources they use to understand how easy it is to reproduce such projects by IoT practitioners around the world.
**Approach.** We analyze the degree of complexity of COVID-19 IoT projects and compare them with those projects that are unrelated to COVID-19. We measure the complexity of IoT projects using the (a) difficulty level, (b) hardware/software/tool items, and (c) the estimated time to reproduce a project. The difficulty level refers to the expertise required by IoT engineers to reproduce a project, such as *beginner*, *intermediate*, *advanced*, or *expert*. The hardware, software, and tools refer to the components required (*i.e.,* cost) to reproduce a project. The estimated time refers to the effort required to reproduce a project. We use Mann–Whitney-Wilcoxon (MWW) rank sum tests (Wilks, 2011) with an *α* = 0.05 to measure how significant the difference is between the complexity of COVID-19 and non-COVID-19 IoT projects. We use Cliff’s delta effect size measure (Cliff, 1993) to verify how significant is the difference in magnitude between the values of two distributions.
**Observation.** ***Developing COVID-19 IoT projects may require a little expertise but can be costly and time consuming to reproduce***. Figure 3A shows the number of COVID-19 and non-COVID-19 IoT projects at different levels of difficulty. We observe that the majority (*i.e.,* over 80%) of both COVID-19 and non-COVID-19 IoT projects are from the beginner and intermediate difficulty levels. Yet, we observe that the percentage of advanced and expert-level COVID-19 IoT is more than that of non-COVID-19 IoT projects. In addition, Fig. 3B shows that COVID-19 IoT projects require more components to be purchased than non-COVID-19 IoT projects in order to reproduce the projects. In particular, the number of hardware items to reproduce COVID-19 IoT projects is significantly more than those for non-COVID-19 IoT projects (*p* − *value* < 0.0001 and *delta* = 0.24, *i.e.,* a small effect size). However, despite the significant difference between the numbers of software items and tools of COVID-19 and non-COVID-19 IoT projects, we observe that the effect sizes are negligible (*delta* values of 0.12 and 0.15, respectively). Moreover, we observe from Fig. 3C that reproducing COVID-19 IoT projects requires significantly more time (a median difference of one hour) than reproducing non-COVID-19 IoT projects. Yet, the effect size is negligible (*delta* = 0.14).
  **Conclusion.** Our results indicate that, although COVID-19 IoT projects mostly require little expertise, reproducing the projects by other IoT engineers may require a bit more effort and time in comparison with other non-COVID-19 IoT projects.

### RQ_4_ : Is the IoT response to the COVID-19 pandemic nation-dependent?

**Motivation.** The impact of the COVID-19 pandemic has been different from one nation to another. The response to the pandemic in some countries has been prompt and strict, whereas the response of other countries has taken much longer. In this RQ, we investigate whether the impact of the pandemic on a certain country yields an analogous impact on the IoT technology.
We address this RQ by studying (i) the leading countries in developing COVID-19 IoT solutions and (ii) whether the number of projects developed is correlated with the number of reported COVID-19 infected cases of the countries identified in our dataset. The approaches and results of this RQ are as follows.

### RQ_4.1_ : Which locations are COVID-19 IoT solutions mostly developed from?

**Approach.** Every project hosted on Hackster.io is developed by one or more IoT engineers. IoT engineers maintain online profiles on Hackster.io, which may include their locations. We crawl the profiles of the owners of COVID-19 IoT projects to identify their locations. IoT engineers of about half of COVID-19 projects shared their geographical locations. We take into consideration the projects that may be developed by IoT engineers from different locations. Then, we group the projects according to the location of their project owners and count the number of projects per each location (at the country level).
**Observation.** ***COVID-19 IoT projects are developed from 46 different countries.*** We observe that the majority (143) of COVID-19 IoT projects are developed by IoT engineers who reside in India, followed by the USA (69) then the UK (26), China (17), Germany (16), Spain (12), Italy (10) and Mexico, Indonesia, and Bangladesh (9 each).

### RQ_4.2_ : Are COVID-19 IoT projects correlated with the reported COVID-19 infected cases?

**Approach.** To the number of COVID-19 IoT projects and COVID-19 infected cases of the countries identified in our dataset, we use the Pearson product-moment correlation coefficient (*r*) (Stigler, 1986). We obtain the total number of reported COVID-19 cases of each country in 2020 using an online repository (https://ourworldindata.org/covid-cases) that has been used in the literature (Buonsenso et al., 2021).
**Observation.** *The number of developed COVID-19 IoT projects is significantly correlated with the number of COVID-19 infected cases*. We observe that there is a significant correlation with a Pearson’s coefficient *R* = 0.52 and *p* − *value* < 0.0001 (a positive relationship that produces an upward slope on the scatter plot). The positive correlation coefficient indicates that when the value the more COVID-19 infected cases a country observes, the more IoT projects to be engineered in that country.
  **Conclusion.** The wide spread of the virus worldwide has a correlated rise in the demand for health-related IoT technologies in the countries that have been affected by the pandemic (https://iot-analytics.com/the-impact-of-COVID-19-on-the-internet-of-things). Countries should pay more attention to the role of IoT technology in tackling this pandemic.

### RQ_5_ : How popular are COVID-19 IoT projects?

**Motivation.** While we observe that IoT engineers have been active to produce IoT solutions to address the COVID-19 pandemic, it is unclear whether such projects receive satisfactory attention from the community members. In this RQ, we investigate whether the COVID-19 IoT projects gain acceptable popularity in comparison with the other IoT projects on Hackster.io. Understanding the popularity measures of COVID-19 IoT projects helps IoT engineers to improve the quality of their projects and produce more useful projects.
**Approach.** To measure the popularity of COVID-19 IoT projects, we collect the number of views (*i.e.,* the number of people who have viewed the project page) and the number of likes or thumbs-up (*i.e.,* user satisfaction) of each project from Hackster.io. Considering that the views and likes is dependent on the time a project was published (*i.e.,* older projects are likely to have more views/likes), we perform our analyses using the average number of views and likes per day. To do this, we divide the total number of views and likes of each project by the number of days since the project was published on Hackster.io. We use Mann–Whitney-Wilcoxon (MWW) rank sum tests with an *α* = 0.05 and Cliff’s delta to measure how significant the differences are between the views and likes of COVID-19 and non-COVID-19 IoT projects.
**Observation.** ***COVID-19 IoT projects are more popular than other IoT projects.*** We find that COVID-19 projects have 2*x* significantly more views per day than non-COVID-19 projects, with a *p* − *value* < 0.0001 and a small effect size (*delta* = 0.29). In particular, the median number of views per day of COVID-19 projects is 9, whereas non-COVID-19 IoT projects have a median of four views per day. Moreover, we find that COVID-19 projects receive 3.5*x* significantly more likes per day than non-COVID-19 projects, with a *p* − *value* < 0.0001 and a medium effect size (*delta* = 0.38).
  **Conclusion.** Our results indicate that COVID-19 IoT projects are perceiving more attention by the IoT community than non-COVID-19 IoT projects, which by itself gives IoT engineers support to continue their work towards defeating the coronavirus.

### RQ_6_ : What technologies do COVID-19 IoT projects use in common?

**Figure 4:**
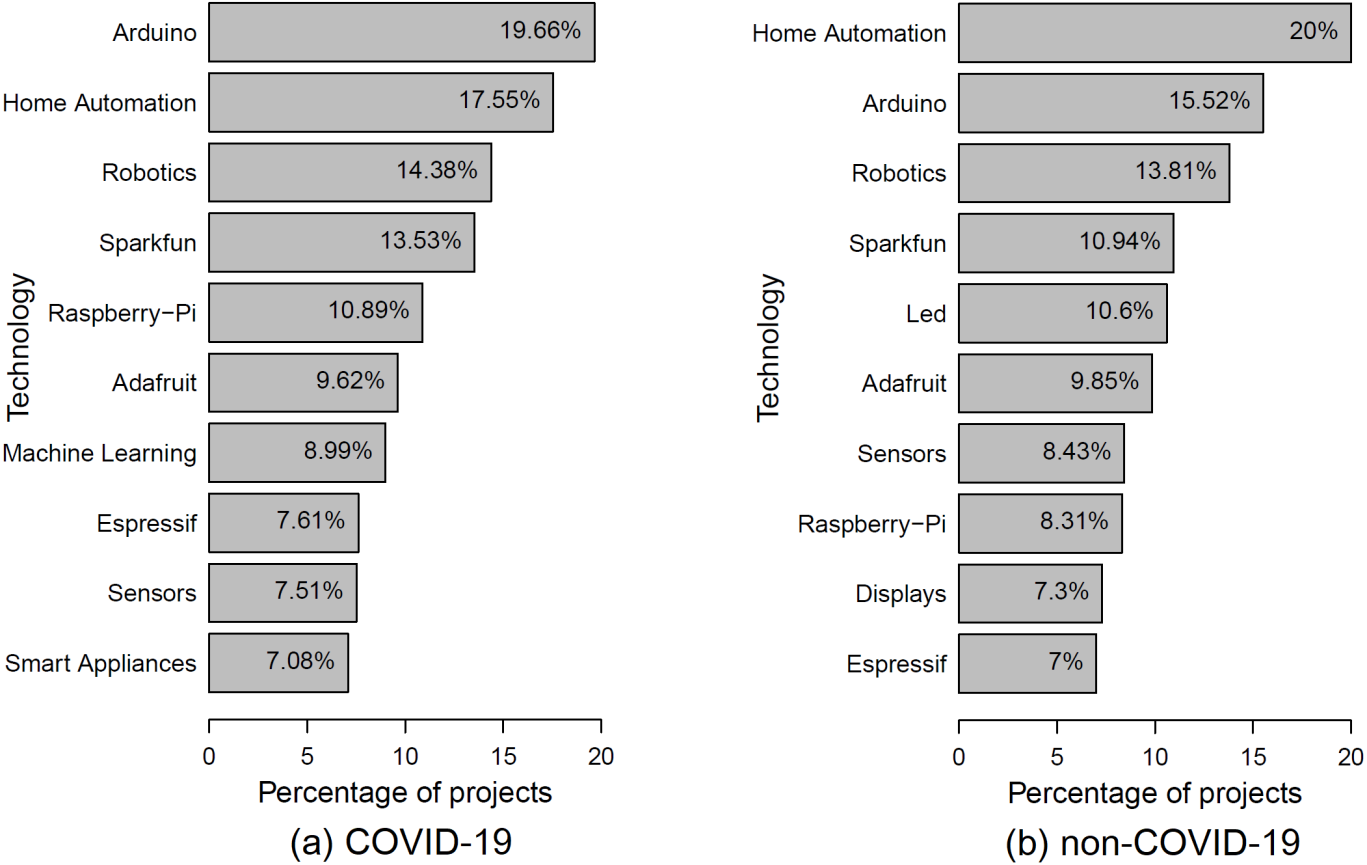
Top 10 technologies of C ovid-19 IoT projects along with their percentages.

**Motivation.** The IoT domain is full of technologies and platforms that ease the life of IoT engineers and make them more productive. In this RQ, we explore the common and most prominent technologies used in COVID-19 IoT projects. Understanding such technologies helps to open more business opportunities and motivate the IoT industry to mass-produce their products and export them to more affected nations.
**Approach.** To identify the commonly used technologies and platforms in COVID-19 IoT projects, we analyze the channels connected to each project hosted on Hackster.io. Channels may not always be technical (*e.g.*, a channel may refer to a community or topic). Hence, in our analysis of the technologies and platforms used by COVID-19 IoT projects, we consider only channels that represent software and hardware platforms. For example, from the top-10 technologies, we exclude the *COVID-19*, *IoT*, and *Health* channels.
**Observation.** ***Most commonly used hardware technologies/platforms by COVID-19 IoT projects disagree with those of non-COVID-19 IoT projects***. Figure 4 shows the top-10 technologies used by IoT projects in Hackster.io. We observe that the most top-10 commonly used hardware technologies of COVID-19 projects, such as *Arduino* and *Robotics*, are not very different from those of non-COVID-19 projects. This result indicates that the requirements to develop IoT solutions to fight COVID-19 are accessible by those whoever is interested in defeating the virus. In addition, we observe that *Machine Learning* tends to be more adopted by COVID-19 projects than those of non-COVID-19 projects.
  **Conclusion.** Our results encourage the hardware industry to take into account the high demand of the hardware platforms required to develop IoT solutions for COVID-19. In addition, IoT engineers are encouraged to collaborate with software and data engineers to developer more advanced COVID-19 solutions.

## Discussion

This section discusses the implications of our results for IoT practitioners, researchers, and industry.

### Acting proactively and continuously to pandemics

Our results show that IoT practitioners started to actively act towards handling COVID-19 in *March* 2020 (*i.e.,* after COVID-19 has been characterized as a pandemic by WHO). While this reaction has been appreciated by the community, IoT engineers should have responded earlier (*e.g.*, as early as *January* 2020) before the situation becomes more complicated, in terms of the possibly imposed restrictions and the increased need to healthcare systems. Moreover, our results show that the number of developed COVID-19 related IoT projects started to decline after many countries have lifted the restrictions in *May* IoT practitioners should realize that a second wave (or even a third wave) of pandemics is highly likely (Mamelund, 2018). A subsequent wave of COVID-19 may require different kinds of IoT solutions/technologies. Hence, IoT engineers and industry should be prepared to make IoT solutions available to their societies beforehand.

### Experience from other domains can improve the IoT solutions for pandemics

Our results show that many IoT solutions have been developed to protect, track, diagnose, and entertain individuals. However, IoT practitioners may be less aware of the consequences of their developed IoT solutions and, hence, need to acquire knowledge of other domains (Chattopadhyay et al., 2015). For example, IoT projects may threaten the privacy of users or may be prone to security vulnerabilities or environmental pollution. In addition, the IoT community may need to forecast future demands for defeating COVID-19. For example, even with COVID-19 vaccines, people may still need IoT solutions to monitor the possible side-effects of such vaccines. Therefore, IoT engineers should work closely with researchers and professionals from other health/technological domains to meet the current and future IoT needs of their societies.

### More easy-to-reproduce COVID-19 related IoT solutions to enable mass production

We observe from our dataset that the median number of COVID-19 IoT project owners is one. We speculate that the complexity observed in the COVID-19 IoT projects is due to the single-author project development. IoT engineers should realize that collaborative work can simplify the design and implementation of the IoT solution, especially with the availability of IoT collaborative framework for advanced manufacturing (Lu & Cecil, 2016). In addition, IoT engineers should be more careful about reporting better estimates of the cost and time required to reproduce their projects Bad estimates may give wrong indications to other IoT practitioners, which may either discourage them from reproducing a project or turn into a situation where reproducing a project is not possible for them.

### Drawing attention to an IoT project can be as important as developing the project

Our results reveal that IoT projects that address COVID-19 concerns have received better popularity (*i.e.,* more views and likes) by the surrounding community. However, we observe that over a third (*i.e.,*  38% of COVID-19 IoT projects do not have the ‘COVID’ or ‘CORONA’ terms in their title, description, and connected tags and channels. Not including such highly associated words to COVID-19 may make those unreachable to customers or other IoT practitioners who might need them. Therefore, we encourage IoT engineers to provide as much information as possible to the web pages of IoT projects to improve spreading the word about the capabilities of the projects.

### IoT industry should work in hand with online IoT communities to promote the most employed technologies for COVID-19

According to a survey conducted by Hackster.io (https://www.hackster.io/survey), the most commonly used hardware platform in IoT projects is *Arduino*. This observation holds in our studied projects (both COVID-19 and non-COVID-19 projects) as *Arduino* is among the top technologies used by the projects in our dataset. Hence, the hardware industry should pay attention to the hardware demand by IoT solutions that handle a pandemic to make them accessible by the IoT community worldwide.

## Related Work

In this section, we present the existing work that conducts studies on pandemics and IoT solutions.

### Pandemic studies

The influence of the COVID-19 pandemic has extended to numerous human activities over the world. Simultaneously, this motivates researchers and scientists to consider this pandemic from different fields and approaches to address its epidemiological results. Ralph et al. (2020) focused on explaining the pandemic effects on developers’ productivity *via* surveying over 2*k* developers using 12 different human languages. Participants feedback indicated that the pandemic has had a negative effect on developers’ productivity and wellbeing. Another study by He et al. (2020) proposed a new comprehensive study of mobile malware that can be hidden under coronavirus apps. The authors observed that the number of mobile apps increased proportionally with the increase of COVID-19 cases over the world. However, about half of the apps are not marked as approved applications, where misleading icons were used to confuse consumers. Another study to analyze Android apps was conducted by Samhi et al. (2021) to study the characteristics of mobile apps that address COVID-19. The study showed that Android apps provided consumers with digital methods, such as health notes, reporting, and distributing data on infected cases, as well as user tracking of infected cases.

### IoT studies

Researchers have studied IoT in a wide variety of problems, *i.e.,* context-aware IoT approaches, fault-tolerance in IoT services, IoT for cloud computing, IoT service composition, and the popularity of IoT projects.

Regarding context-aware approaches, Chattopadhyay et al. (2015) presented an analytical method that helps engineers to build IoT applications without the need to have heavy knowledge of signal processing or any other specific domains. D’Oca & Hong (2014) proposed a framework with two data mining techniques (*i.e.,* clustering and associated rules) to identify the behavior of occupants related to the opening and closing of windows. The authors found that indoor air temperature, outdoor air temperature, and the presence of occupants were the most important factors for opening/closing windows. Regarding fault tolerance in IoT services, Su et al. (2014) proposed an approach that allows the achievement of *failover* mechanisms upon the replacement of IoT devices. Their results show that failures may be recovered within seconds without the need for human interference.

With respect to the IoT and cloud computing integration, Botta et al. (2014) conducted a literature review to understand the potential applications and challenges of using IoT and cloud computing together (*i.e.,* the *CloudIoT* paradigm). The authors identify several open issues, such as the need for more standardization in both IoT and cloud computing fields. With respect to IoT composition, Tzortzis & Spyrou (2016) proposed a semi-automatic approach that allows project owners to discover, consume, and interconnect IoT services to create more complex services. They evaluate their approach by interconnecting simple IoT-enabled services. Ustek-Spilda et al. (2020) studied how active are social media (in particular, Twitter) discussions about the IoT technology in Europe. The authors found that users from the same geographical context are more likely to be connected online than users from different geographical contexts. The authors also observed that IoT-related hashtags(*e.g.*, #healthcare, #hardware, #IoT, and #startups) are highly correlated. Lampropoulos, Siakas & Anastasiadis (2018) presented a study of the different IoT applications in terms of industrial fields such as transportation and communications, healthcare and sanitation, smart cities, smart ecosystems, and manufacturing. Zanella et al. (2014) also presented an urban IoT system that concentrates on the concept of the Smart City vision, which seeks to take advantage of the most modern connectivity technology to support value-added systems for city government and for residents.

One of the most common IoT-adopted sectors is the medical industry. Using IoT technology, new opportunities, programs and software will be developed to enhance the healthcare and healthcare industry. For example, Gómez, Oviedo & Zhuma (2016) developed an ontology-based architecture for fitness and exercise management to offer guidelines for patients with chronic diseases. Ghaleb, Da Costa & Zou (2021) studied the the important characteristics of popular IoT projects on Hackster.io to allow IoT engineers understand how to improve the popularity of their projects to attract more users and foster business opportunities.

Unlike the aforementioned work, our study focuses on the role of IoT technology to handle the COVID-19 pandemic.

### IoT solutions addressing pandemics

Sareen, Sood & Gupta (2018) conducted a comprehensive review of the possible IoT solutions that address the COVID-19-like viruses, such as Middle East Respiratory Syndrome(MERS) and Severe Acute Respiratory Syndrome (SARS). The authors provided a guideline for leveraging existing IoT solutions to meet societal needs and help in reviving from pandemics. For the diagnosis and monitoring of Ebola infected individuals, Sareen, Sood & Gupta (2018) proposed a novel framework based on Radio Frequency Identification Device (RFID), wearable sensor technologies, and cloud computing infrastructure. Zhu et al. (2020) proposed a Polymerase Chain Reaction (PCR) device that can be used to detect pandemic diseases and track their progress. Such a device is portable and user friendly, which enables users to connect through Bluetooth to Android devices. The system was used to evaluate the complementary DNA (cDNA) of the Dengue virus(DENV), which can also be used to detect RNA. The device can also collect geographical locations, which allows officials to spot the spread of diseases. Meraj et al. (2021) investigated IoT solutions developed to detect and predict infectious diseases, such as the flu, Zikaas, H1N1, well as COVID-19. The authors proposed a solution that places a set of sensors throughout a workplace to detect or collect information about infectious diseases.

### IoT solutions for the COVID-19 pandemic

IoT plays a vital role in the battle against COVID-19. There have been many solutions that were established to handle safety and protection precautions related to COVID-19.

Nasajpour et al. (2020) explores the impact of IoT-based solutions in COVID-19 pandemic and investigates state-of-the-art designs, technologies, applications, and industrial IoT-based solutions for diagnosis, quarantine, and recovery. Mohammed et al. (2020a) introduced a smart helmet-mounted device with thermal and face recognition to recognize infected people among the crowd. Mohammed et al. (2020b) also proposed a drone-based technology to speed up locating infected persons and places. Yang et al. (2020a) proposed a safety solution to assist health workers and decrease the stress level when treating patients. Yang et al. (2020b) also integrated the Internet of Medical Things (IoMT) with smartphone capabilities, such Geographic Positioning System (GPS), to track infected cases and provide home treatment. Singh et al. (2020) highlighted twelve IoT applications to discover symptoms of COVID-19 and provide better protection against the COVID-19 pandemic. (Chamola et al., 2020) studied the impact of COVID-19 on the global economy and explored how different technologies, including IoT, are used to mitigate such an impact. Kumar, Kumar & Shah (2020) reviewed existing COVID-19 IoT techniques and suggested an IoT-based architecture to minimize the spreading of COVID-19. Ndiaye et al. (2020) studied how COVID-19 affected the evolution IoT technologies and how sensor networks could be useful in the future to address global pandemics.

Fatima et al. (2020) proposed an IoT fuzzy inference framework to effectively predict and monitor COVID-19. The proposed framework contained three layers, including sensory, preprocessing, and application layers for data collection, data cleaning, and prediction. Allam & Jones (2020) emphasized the critical necessity to standardize the protocols for improved smart city communication and the need to democratize the smart city technology sector to foster equality and transparency among stakeholders, which allows for more collaboration against disasters, in general, and COVID-19, in particular. Senturk et al. (2020) used cloud computing techniques to estimate the number of persons which causes pollution in the transportation. To determine the threat level of COVID-19 infections for each transportation, the collected data was investigated systematically and historically. Deep learning models were employed by Iskanderani et al. (2021) to automate the diagnosis of COVID-19 using chest X-rays acquired using medical sensors. Akbarzadeh, Baradaran & Khosravi (2021) designed a smart hospital by utilizing sensors to manage the number of visitors and occupied areas they occupy.

All the above studies did not explore the IoT solutions provided by the community to the community but rather focused on the industrial solutions. Also, little is known about the open source IoT technologies that enable people to build their own devices to defeat COVID-19. Our study sheds light on how actively the open source IoT technology tackles the COVID-19 pandemic, the characteristics of those projects, the technologies being used, and how are they perceived by the surrounding community.

## Threats to Validity

This section discusses the potential threats to the validity of our work.

### Construct validity

Construct threats to validity are concerned with the degree to which our analyses measure what we claim to analyze (Shull, Singer & Sjøberg, 2007). Mistakenly computed values may influence our conclusions. For example, the (sub)categories of COVID-19 IoT projects extracted are restricted to a sample of 274 out of 946 projects (*i.e.,* more projects could produce more (sub)categories). We mitigate this threat by making sure that (a) our random sample is statistically significant (a confidence level of 95% and a confidence interval of ±5%), (b) two co-authors have independently labeled the data, and (c) disagreements have been resolved by a third co-author. Yet, future work should extend our identified (sub)categories by performing manual analyses on a more large-scale sample of projects. In addition, the textual description of project could be misleading and might not reflect what the project actually does. To mitigate this issues, we also investigated the comments raised by community members to see if someone is raising an issue regarding false information about that project. We found no cases in which a project is identified to provide false information. We were unable to analyze the code of projects, since (a) not all projects have code associated with hardware devices, (b) some projects do not share their code, and (c) code could be written using different programming languages.

### Internal validity

Internal threats to validity are concerned with the ability to draw conclusions from the attributes of the projects in our dataset (Shull, Singer & Sjøberg, 2007). We employ a set of COVID-19 related keywords to identify the IoT projects that address COVID-19 in particular. We realize that our keywords may include COVID-19 unrelated projects or may miss some projects that are COVID-19 related. To mitigate this threat, we run our search in multiple iterations, starting from an expanded set of keywords and excluding the keywords that generate high numbers of COVID-19 unrelated projects after manually analyzing a sample of the resulting projects in every iteration.

### External validity

External threats are concerned with our ability to generalize our results (Shull, Singer & Sjøberg, 2007). Given that our study is limited to a single online community that hosts IoT projects (*i.e.,* Hackster.io), we cannot generalize our conclusions to IoT projects in other online communities. To mitigate this issue, we investigate other websites, such as I nstructables and H ackADay. However, we find that, unlike other websites, Hackster.io is dedicated to complete projects that are developed to achieve a specific job. Other websites are too broad in the sense that IoT engineers and companies can even publish hardware and software or training courses. In addition, unlike other websites, Hackster.io provides additional information for the IoT community to distinguish between good and bad projects, such as showing the estimated time to reproduce a project, respects received by a project, difficulty level of the project, the amount of instructions provided by project owners. Also, given the variation and inconsistency in the design and structure of websites, each website would need a customized crawler. Still, future work should investigate whether our observations may hold for projects published in other online communities.

## Conclusion

In this paper, we conduct an empirical study to investigate the role of the IoT technology in responding to the COVID-19 pandemic. In particular, we study the Internet of Things (IoT) solutions that have been developed to deal with COVID-19. To this end, we collected data about IoT projects hosted on Hackster.io to understand the role of the IoT technology in helping the community. Unlike other websites, Hackster.io is an IoT community dedicated to completed IoT solutions and provides community members with information about the estimated time to reproduce a project, project popularity, its difficulty level, and the amount of instructions provided by project owners. We performed analyses to study (1) how active is the IoT community in responding to the COVID-19 pandemic, (2) the aspects of COVID-19 addressed by IoT projects, (3) the complexity of COVID-19 IoT projects, (4) nation-dependent analysis of COVID-19 IoT projects, (5) the popularity of COVID-19 IoT projects, and (6) the technologies/platforms that COVID-19 IoT projects employ. We also reviewed previous IoT solutions that were proposed to address pandemics in general and COVID-19 in particular. The key findings of our study are (a) IoT engineers actively work towards providing solutions to help fight the pandemic; (b) COVID-19 IoT projects address *protection*, *diagnosing*, *tracking*, lockdown entertaining; (c) COVID-19 IoT projects may require little expertise but can be slightly more costly and time-consuming to reproduce; (d) the more COVID-19 infected cases in a country, the more COVID-19 IoT projects to be developed; (e) COVID-19 IoT projects are more popular (*i.e.,* more views and likes) than other IoT projects; and (f) most commonly used hardware technologies/platforms by COVID-19 IoT projects are not far from those of non-COVID-19 IoT projects.

Our findings encourage IoT engineers, researchers, and practitioners to develop more projects and conduct further experiments to support the precautions provided by WHO to handle the COVID-19 pandemic. Future work should consider two directions for empirical research: (i) explore the geographical, technical, and economical challenges to develop IoT solutions for COVID-19, such as interoperability, security & privacy, as well as the impact on the IoT and health industries. (ii) support our findings by considering other sources of information, such as surveying IoT engineers and practitioners to investigate their needs and challenges to provide IoT solutions, in addition to investigating additional online IoT communities.

## Supplemental Information

10.7717/peerj-cs.776/supp-1 Supplemental Information 1 A taxonomy of COVID-19 IoT projects

10.7717/peerj-cs.776/supp-2 Supplemental Information 2 Dataset used for the empirical analyses

## Additional Information and Declarations

### Data Availability

The following information was supplied regarding data availability: The data used in our analyses are available in Supplemental File.
