# Supplementary figures and images for: How Internet of Things responds to the COVID-19 pandemic
Source: PeerJ Comput Sci. 2021 Nov 10;7:e776. doi: 10.7717/peerj-cs.776 (PMC8592249; doi:10.7717/peerj-cs.776)

**COVID-19  
IoT Solutions**

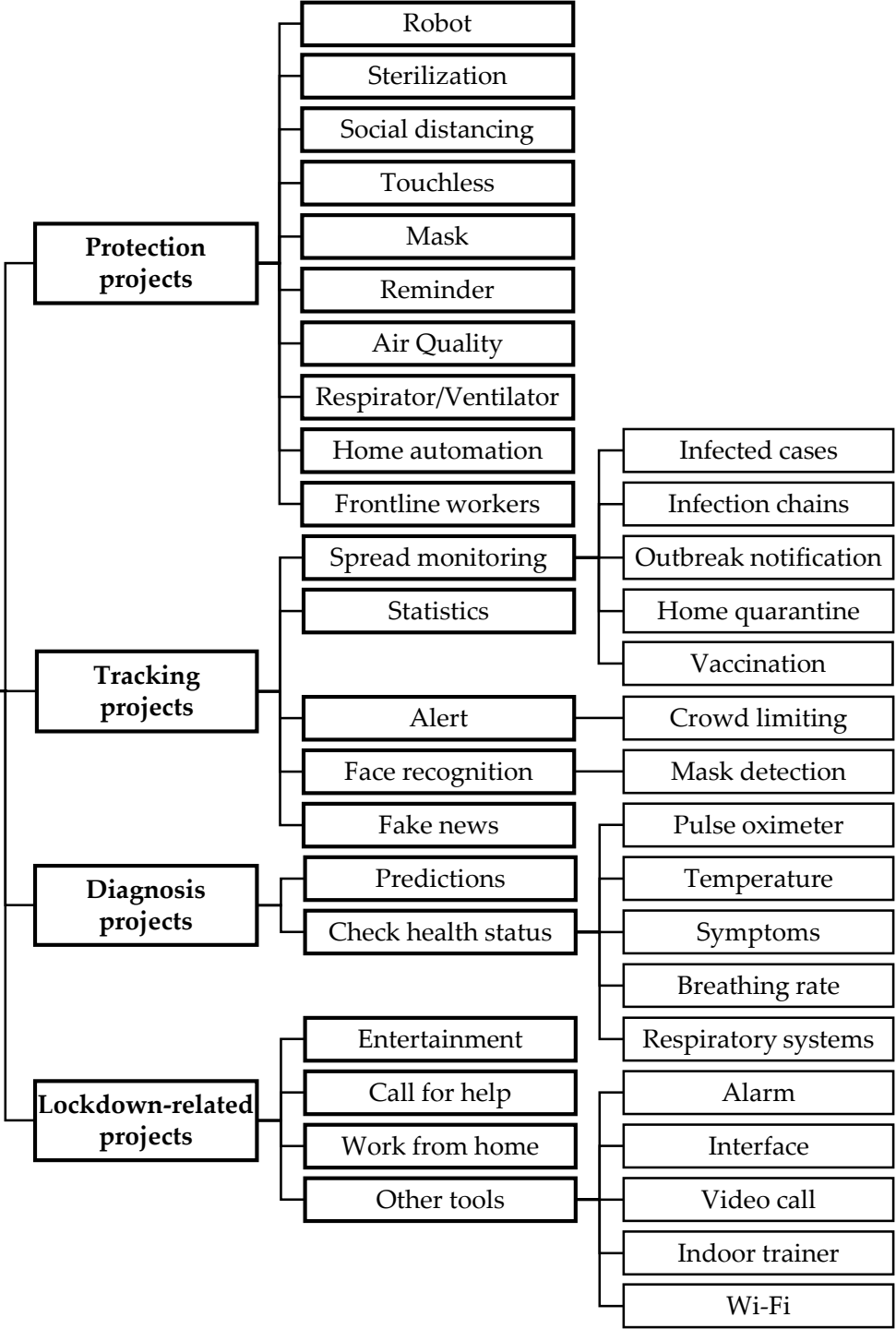

Supplement: Supplemental Information 1 [file peerj-cs-07-776-s001.pdf]
